# Taxonomy Meets Neurology, the Case of Amyotrophic Lateral Sclerosis

**DOI:** 10.3389/fnins.2018.00673

**Published:** 2018-09-26

**Authors:** Giovanna Morello, Antonio Gianmaria Spampinato, Francesca Luisa Conforti, Sebastiano Cavallaro

**Affiliations:** Institute of Neurological Sciences, Italian National Research Council, Catania, Italy

**Keywords:** ALS, amyotrophic lateral sclerosis, molecular taxonomy, personalized medicine, neurodegenerative disease, multiomic analysis, signaling pathways

## Abstract

Recent landmark publications from our research group outline a transformative approach to defining, studying and treating amyotrophic lateral sclerosis (ALS). Rather than approaching ALS as a single entity, we advocate targeting therapies to distinct “clusters” of patients based on their specific genomic and molecular features. Our findings point to the existence of a molecular taxonomy for ALS, bringing us a step closer to the establishment of a precision medicine approach in neurology practice.

“Examining the genetic components of more common and—complex illnesses will require locating and analyzing the structure and coordinated action of groups of genes [.] to developing methods for early diagnosis and treatment.”

(*Charles DeLisi, The Human Genome Project, 1988) (DeLisi, 1988*).

During the mid-1980s, three scientists independently and nearly simultaneously came up with the idea of sequencing the entire human genome: Robert Sinsheimer, then Chancellor of the University of California–Santa Cruz (UCSC), Salk Institute researcher Renato Dulbecco and Charles DeLisi of the United States Department of Energy. Their visions and perspectives marked a turning point in the history of medical diagnosis and therapy, paving the way for a new era of healthcare, the era of “personalized medicine.”

Traditionally, diagnostic practice relied upon assessing physical signs and symptoms or on physiological and biochemical alterations that, despite providing valuable information about clinical course, are often not sufficient to fully characterize the complexity and heterogeneity underlying molecular mechanisms in different individuals. As a result, drugs continue to be ineffective in a large proportion of patients, while they cause side effects, or may result in overall treatment effects that are clinically insignificant, leading to the conclusion of minimal effectiveness.

The paradigm of precision medicine, as introduced by former US President Barack Obama in his 2015 State of the Union address, aims to overcome the “one-size-fits-all” approach, moving toward the use of individual unique patient's molecular profiles to optimize and individualize patient care, enabling the provision of *the right therapeutic strategy*, *for the right person, at the right time* (Thibaut, 2016). This concept involves the development of a new and more accurate classification of individuals into subpopulations that differ in their predispositions to a particular disease, in the molecular underpinning of the disease they develop and/or in their response to a specific treatment (Figure 1). This new taxonomy brought radical changes in the way we think about human diseases and provided new information for predicting clinical outcomes and novel strategies to treat diseases in a dedicated, mechanism-specific way.

**Figure 1:**
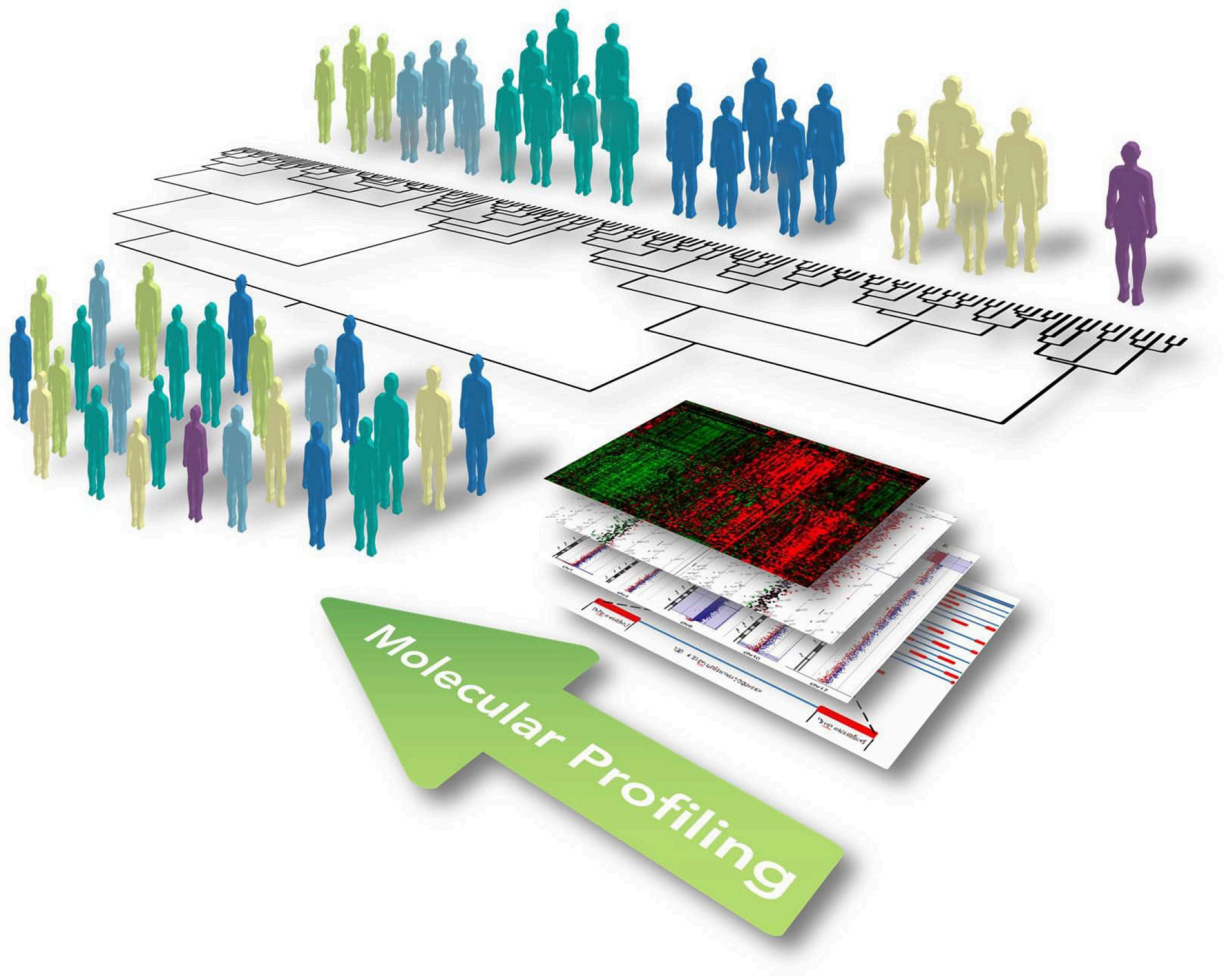
Illustrative representation showing the concept of functional and integrated multi-omic approach for optimizing patient stratification and precision medicine. Widescreen genomic profiling of complex and multifactorial disorders, including ALS, has allowed us to better define disease mechanisms in different patients and identify multiple biomarkers on the gene, protein, and genomic scale. The omics profile of each individual, including the genome, transcriptome, proteome, and metabolome, should be eventually linked up with phenotypes obtained from clinical observations, medical images, and physiological signals. This information can be subsequently incorporated together using computational strategies to identify causal disease networks, stratify patients, and ultimately allow progression toward precision medicine encompassing new and selective therapeutics and preventative therapy.

The prospect for building a “New Taxonomy” of diseases and realizing the promise of precision medicine has been dramatically improved by the significant advancement of high-throughput technologies (such as genomics, transcriptomics, proteomics, metabolomics), and diagnostic techniques throughout the past decades. Construction of a standardized model for the integrated analysis and interpretation of such multiple heterogeneous data types in the context of complex molecular pathways and networks is an imperative requirement to improve diagnostic accuracy and to optimize therapeutic strategies based on specific molecular profiles of diseases (Figure 1). In this context, data-driven clustering using statistical methods represents one of the commonest ways to identify disorder-specific features among high-dimensional big data for an improved taxonomy of human diseases (Kristensen et al., 2014; Figure 1). This approach is based on the basic principle from which genes with the same interaction partners or expression data, most likely share the same biological function. However, in the multi-dimensional interaction scenario of a complex human disease, more advanced computational methods (i.e., machine learning approach, network-based methods and Bayesian analysis) are required, with the aim to integrate information from each level of the omics spectrum in a layered network that exchanges information within and between layers, developing a mechanistic understanding of the behavior of different molecular components within biological systems and identifying coherent biological signature associated to phenotypic (Huang et al., 2017).

Oncology represents the first and the most well-characterized example of the application of this molecular-based taxonomy paradigm to daily practice, with a multitude of successful individualized or customized therapeutic strategies and immunotherapies available for a wide range of cancers. Key examples include glioblastoma, breast, and colon carcinoma, for which have been developed commercially available assays (i.e., MammaPrint70-gene signature or KRAS mutational analyses) able to subdivide patients into selective clinically relevant sub-types characterized by different genetic alterations, genomic profiles and prognostic biomarkers.

Despite considerable progress in cancer and other therapeutic areas, including cardiovascular, rheumatic, and neuromuscular diseases, precision medicine remains an aspirational objective when dealing with multifaceted heterogeneous clinical states such as neurodegenerative disorders, which may reflect combined effects of various genes and their interaction with environmental factors. Because of their heterogeneous and multifactorial etiology and the limited accessibility to post-mortem tissues, knowledge about molecular underpinnings leading to the majority of neurological diseases is still incomplete, and translation of current data into tailored clinical applications remains a challenging task.

In the last years, our research group has taken important steps toward the development of a new approach to the classification of neurodegenerative diseases, highlighting for the first time the existence of a biological and molecular heterogeneity of amyotrophic lateral sclerosis (ALS), a severe clinical condition characterized by upper and lower motor neuron degeneration for which there is no truly effective treatment. Since the discovery of *SOD1* mutations in familial ALS cases (fALS) in 1993, ALS research has in fact entered a molecular genetics era, revealing ALS as a complex disease characterized by a high degree of genetic heterogeneity in which a constellation of causative genes and risk factors have been identified (Conforti et al., 2012). The complexity of its genetic architecture, including an important role for rare genetic variants, has completely transformed the way we think about ALS, leading us to reconsider the traditional classification system. Hence, taking into account the genomic background of ALS, we performed a comprehensive transcriptional analysis of motor cortex samples from control and sporadic ALS (SALS) patients, grouping these on the basis of their similarities measured over the most “hypervariable genes” (9.646 genes with a standard deviation >1.5). Unsupervised hierarchical clustering analysis allowed to discriminate controls from SALS patients and clearly distinguished two greatly divergent SALS subtypes, each associated with differentially expressed genes and pathways (Aronica et al., 2015). The same accurate transcriptome-based classification was confirmed by utilizing just a restricted subset of genes extensively implicated in monogenic forms of ALS, suggesting that sporadic and monogenic forms of ALS share common etiopathogenic mechanisms and confirming the existence of a molecular heterogeneity in ALS (Morello et al., 2017a). These findings are not completely surprising if we consider that clinical studies on edaravone, a free radical scavenger recently approved by FDA for ALS treatment showed effectives only in specific sub-cohorts of patients, supporting the need for clinical trials to take individual variability and genotypic features into account (Hardiman and van den Berg, 2017; Katyal and Govindarajan, 2017). To this regard, our analyses also revealed a number of potential biomarkers and therapeutic targets that were differentially deregulated in specific subsets of ALS patients and exhibited expression patterns similar those of the ALS animal models at different disease stages, offering a useful starting point for the further development of personalized diagnostics and targeted therapeutic plans (Morello and Cavallaro, 2015; Apolloni et al., 2017; Morello et al., 2017a,b,c). Another interesting application being investigated is attempting to determine whether transcriptional alterations in SALS may be related to genomic DNA alterations, offering the potential to define disease subgroups and their molecular signatures at yet another level of systemic complexity. These findings may hold profound implications both for identification of etiopathogenic mechanisms that were not put in evidence by considering ALS pathology as a single entity, but also for the individualization of diagnostic and therapeutic approaches, bringing us a step closer to the establishment of a more efficacious and personalized genome-guided medicine for ALS.

In the context of a personalized medicine initiative, there is considerable interest in developing accurate biomarkers to detect pathophysiologic processes while still latent, providing the greatest opportunity for effective therapeutic intervention. To this regard, the combination of clinical and genomic data with structural/functional/metabolic neuroimaging techniques as well as biofluid (i.e., saliva, cerebrospinal fluid-CSF-and blood) analysis, offers a valuable resource to increase overall power and accessibility to detect previously inaccessible disease-related biomarkers and mechanisms prior to symptom onset. A recent example is represented by the strong association existing between higher CSF and blood levels of phosphorylated neurofilament heavy chain (pNFH) and shorter survival in ALS patients, in particular those carrying C9orf72 expansion, supporting a potential value of this marker for identifying more homogenous patient subgroups and guiding individual treatment decision-making (Gendron et al., 2017). In addition to proteomics, neuroimaging technologies (i.e., magnetic resonance imaging and spectroscopy, diffusion imaging, functional MRI), thanks to their non-invasive properties, have becoming a vital component of the diagnostic workup for ALS and other neurodegenerative conditions, such as Alzheimer's and Parkinson's diseases, facilitating their differential diagnosis and leading to appropriate medications (Wang et al., 2011; Varghese et al., 2013; Rispoli et al., 2018). However, the development of viable diagnostic, prognostic and disease progression markers at an individual level remains to date as one of the primary challenges of ALS research. Along this line, highly coordinated and multicentric research programs (i.e., Project MinE USA, Answer ALS and the New York Genome Center) are underway, promoting the development of open-access initiative to collect and combine data across neuroimaging and multi-omics layers from ALS patients, in order to identify the various disease subtypes and specific compounds to treat each (Project MinE ALS Sequencing Consortium, 2018).

In summary, although still in its early stages, the potential of molecular taxonomy in neurology is clear and the hurdles for the employment of this powerful new tool are rapidly being overcome. Looking into the future, we envision that prodigious advances in genomics, proteomics and biomarker development for ALS and other complex disorders may combine with the standard classification system, enriching books of neurology with the wholly new vocabulary of genomic and personalized medicine.

## Author contributions

GM and AS wrote the manuscript. FC participated in revising the manuscripts. SC conceived, directed, and supervised the project. All authors have read and approved the final version of this manuscript and agreed to be accountable for all aspects of the work.

### Conflict of interest statement

The authors declare that the research was conducted in the absence of any commercial or financial relationships that could be construed as a potential conflict of interest.
